# Comparative efficacy and safety of traditional Chinese patent medicine for endometriosis: A Bayesian network meta-analysis protocol

**DOI:** 10.1097/MD.0000000000016473

**Published:** 2019-07-19

**Authors:** Shuangqian Dong, Jianwei Zhang, Fengting Zhai, Xinglong Zhao, Xiuyun Qin

**Affiliations:** a The First Clinical College; b Hospital Affiliated to Shandong University of Traditional Chinese Medicine, Jinan; c Traditional Chinese Medical Hospital of Tai’an, Tai’an; d Rizhao Maternal and Child Health Care Hospital, Rizhao, Shandong Province, China.

**Keywords:** endometriosis, network meta-analysis, protocol, traditional Chinese patent medicine

## Abstract

**Background:**

Endometriosis is a common disease of women of childbearing age. In recent years, the incidence of endometriosis has been on the rise. The main clinical manifestations are pelvic pain and infertility. In recent years, traditional Chinese patent medicine (TCMP) has played an important role in the treatment of endometriosis. So far, there is a lack of comparison among all the current common TCPMs for endometriosis. Consequently, it is indispensable to propose a network meta-analysis (NMA) protocol to discuss the strengths and weaknesses of different TCMPs.

**Methods:**

We will comprehensively and systematically retrieve the relevant Chinese and English databases from their inceptions to the May 2019. All randomized controlled trials (RCTs) of TCMPs for Endometriosis will be included. Two researchers will independently screen literature, extract data and assess the risk of bias of included studies. We will conduct pairwise meta-analyses and Bayesian network meta-analyses to assess all the available evidence. Data will be analyzed using STATA and WinBUGS software.

**Results:**

This work will compare and rank the relative efficacy of different TCPMs in the treatment of endometriosis in detail.

**Conclusion:**

The results of this study will provide reliable evidence for the selection of clinical treatment program and guideline development.

**PROSPERO registration number:**

CRD42019127781.

## 1 Introduction

Endometriosis is a nonmalignant, chronic and common gynecological disease defined as the implantation of endometrium in an abnormal or ectopic location outside the uterus.^[1–3]^ The exact prevalence of endometriosis is unclear.^[4]^ It is estimated that it accounts for about 10% to 15% of women in reproductive age around the world,^[5]^ up to 50% of women with infertility.^[4]^ Endometriotic lesions are present mainly in the pelvic including ovaries, pelvic peritoneum, the pouch of Douglas and the rectovaginal septum.^[6]^ It can also be found in the abdominal wall, upper abdomen, diaphragm, bowel, and lungs.^[7]^

This disease is often associated with long-term chronic pelvic pain, infertility, dysmenorrhea, dyschezia, dyspareunia, pelvic-organ dysfunction as well.^[8–10]^ These discomfort symptoms have a negative effect on the quality of women's lives.^[2,11]^ It impacts significantly on psychological and social lives of women across several domains including diagnostic delay and uncertainty, quality of life and everyday activities, education and work, planning for and having children et al;^[10]^ Furthermore, the economic burden on the patient and health care system is massive.^[12]^ In a retrospective cohort study, the direct and indirect costs of endometriosis patients is significantly higher than that of non-endometriosis and, on average, in 12 months, the direct cost per patient increased by $10,000 while the indirect cost increased by $2132.^[13]^

The diagnosis of endometriosis should be based on the history, symptoms, signs, pelvic examination, and auxiliary examination. Auxiliary examinations generally include biomarkers genetics, ultrasound, computerized axial tomography (CT) and magnetic resonance imaging (MRI) and the first choice for the imaging diagnosis of ovarian endometriomas is transvaginal ultrasonography (TVS).^[14]^ The gold standard for diagnosis of endometriosis remains the combination of laparoscopy and the histological verification, but the diagnosis of endometriosis is often delayed for a long time.^[4]^ Research by Nnoaham et al showed that there was a 6.7-year delay between symptoms appear and surgical diagnosis of endometriosis, and a longer delay in health care centers funded by the state (8.3 years vs 5.5 years).^[9]^

Despite a large number of study, the pathogenesis of endometriosis remains unclear.^[15]^ In 1921, Sampson proposed the theory that endometrium tissue is implanted by menstrual blood flowing retrograde into the peritoneal cavity through the fallopian tube.^[16]^ From the perspective of immunology, endometriosis is an inflammatory disease.^[1,17]^ Endometriosis is a “heritable, hormone-dependent” disease according to Nyholt.^[18]^ Moreover, The local inflammatory microenvironment activated by hormonal and immune factors can promote the persistence of endometriosis.^[19]^ Also, the pathogenesis of endometriosis is related to the following factors: Celomic theory, aromatases, deep infiltrating endometriosis, hormonal receptors and epigenetic modulators.^[14]^

The treatment of endometriosis varies according to symptoms, severity of the disease, and the need to maintain fertility.^[20]^ The treatment of endometriosis includes drug therapy, surgical, combination of surgery and medication. Therapeutic drugs include androgens, oral contraceptive pills (OCPs), progestogens, anti-progestogens and gonadotrophin-releasing hormone analogues (GnRH-a).^[21]^ The main purpose of drug therapy is to induce a hypo-estrogenic environment to inhibit the growth of ectopic endometrium.^[21,22]^ But, long-term drug treatment may increase the risk of adverse reactions, such as, headaches, edema, acne and cycle irregularity (related to the use of progesterone), abnormal bleeding, nausea, weight gain and thrombosis (related to the use of oral contraceptives), vaginal bleeding and dryness, hyposexuality, hot flushes, osteoporosis, breast tenderness, insomnia, and depression (related to the use of GnRH agonists).^[21,23]^ Surgical treatment of endometriosis has been proven efficacy, but it is not without any risk.^[24]^ Reoperation, ovarian damage and high recurrence remain huge challenges. It was reported more than half of patients with endometriosis underwent reoperation, and 27% of them needed three or more operations.^[25]^ Severe ovarian damage is not a rare event following surgery, the incidence was 13%.^[26]^ And ovarian reserve is distinctly reduced.^[27]^ The recurrence rate was high, approximately 21.5% at 2 years and 40% to 50% at 5 years after surgery.^[24]^

Traditional Chinese patent medicine (TCPM) plays an important role in treating diseases, reducing disease recurrence and adverse reactions. Many studies and system reviews have confirmed the clinical effect of TCMP for endometriosis. The commonly used TCMPs are Guizhi Fuling pill, Dan’e-fukang soft extract, Sanjie Zhentong capsule, Kuntai capsule, and so on. Liu et al found that Kuntai capsule can effectively reduce the symptoms of local low estrogen induced by GnRH-a and improve the serum BGP level through a randomized controlled trial.^[28]^ A meta-analysis of the efficacy and safety of Dan’e-fukang soft extract in the endometriosis treatment manifested that Dan’e-fukang soft extract was more efficient than gestrinone and the adverse reaction rate was lower than that of western medicines.^[29]^ Due to the variety of TCMPs for the treatment of endometriosis, traditional meta-analysis cannot achieve a comprehensive comparison among different varieties. The purpose of this NMA protocol is to compare the efficacy and safety of different TCMPs in the treatment of endometriosis and provide comprehensive summary evidence.

## 2 Materials and methods

### 2.1 Study registration

We compliant Preferred Reporting Items for Systematic Review and Meta-Analysis Protocols (PRISMA-P) guidelines to conduct this study.^[30]^ This NMA has been registered in the International Prospective Systematic Registration Review (PROSPERO) and the registration number is CRD42019127781.

### 2.2 Inclusion criteria

#### 2.2.1 Type of research

We will include all relevant RCTs of TCPM for endometriosis published in Chinese or English.

#### 2.2.2 Types of patients

The diagnosis of endometriosis will follow the guidelines for endometriosis ^[4]^ or other criteria. Age, race, severity of illness, duration of illness will not be restricted.

#### 2.2.3 Interventions

The treatment group must have been treated with a kind of TCPM in combination with routine Western medicine. If both the 2 groups receive surgical treatment, the surgical treatment must be consistent. The experimental group treated with 2 kinds of TCPM or combined with other TCM treatment methods such as Chinese herbal decoction, Chinese herbal enema, acupuncture, moxibustion and other interventions will be excluded. The use of TCMP is limited to oral administration regardless of course of treatment and dosage.

#### 2.2.4 Outcomes

The primary outcomes include clinical total effective rate, clinical pregnancy rate and pain relief. The secondary outcomes are as follows: recurrence rate, the level of CA125 in serum and adverse events. One or more primary outcome must be covered in the included literature.

### 2.3 Database and search strategy

In this systematic review and NMA, we will comprehensively search the following electronic databases: Cochrane Library, PubMed, Web of Science, EMBASE, Chinese Biomedical Literature Database (SinoMed), Chinese National Knowledge Infrastructure (CNKI), Wanfang database and VIP database from their inceptions to May 2019. The references in the included study will be tracked so as to supplement relevant literatures. We will adjust the basic retrieval strategy according to the characteristics of different databases. The detailed search strategy for PubMed database is shown in Table 1.

**Table 1:**
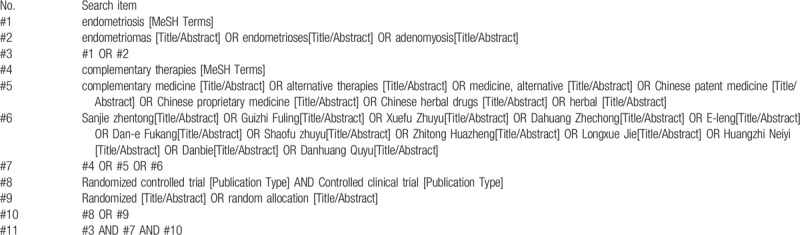
Detailed search strategy for PubMed.

### 2.4 Study selection and data extraction

According to the pre-determined search strategy, the articles retrieved from the above databases will be imported into Endnote x8 for classification and management. Two researchers will independently select studies and extract data. In case of disagreement, it will be settled through discussion or by a third reviewer. The following data information will be recorded: author, title, country, date of publication, journal, random method, registration number; Age, race, duration of disease, stage of disease, diagnostic criteria and sample size; course of treatment, specific interventions and detailed outcome. If the required data is not complete, we plan to contact the original author. If not replied, we will only analysis the available data and explain the possible impact for results of missing data in the discussion section.

### 2.5 Risk of bias assessment

Two researchers will independently evaluate the quality of each included trial according to the Cochrane Risk of Bias Tool recommended by Cochrane Handbook Version 5.1.0. Evaluation criteria includes seven items and each aspect will be categorized as “low” “high” or “unclear”. In the process of evaluation, if there are disagreements, they will be resolved through discussion or a third reviewer.

### 2.6 Statistical analysis

First, we will conduct pairwise meta-analyses for direct evidence. Odds Ratio (OR) will be used for dichotomous data and Mean Difference (MD) or Standardized Mean Difference (SMD) for continuous data. The 95% credible interval (CI) of each effect size will be calculated. We will use I^2^ test to assess statistical heterogeneity.

Then, we will perform a Bayesian NMA using random-effects model to compare the direct and indirect evidence. The posterior probability is inferred according to the prior probability, and estimation and inference will be carried out under the assumption that Markov Chain Monte Carlo (MCMC) has reached stable convergence state. When running WinBUGS program, we will adjust the number of iterations and annealing times according to the characteristics of each outcome database. The surface under the cumulative ranking curve (SUCRA) valued expressed as percentages^[31]^ will be calculated to rank the treatment effect for each intervention. The node splitting method will be used to evaluate the consistency of indirect and direct comparisons if there is a “closed loop”.

### 2.7 Subgroup analysis

If there is enough evidence, we will conduct subgroup analysis to explore the sources of heterogeneity. The following aspects will be used: age, surgical treatment or not, course of treatment**.**

### 2.8 Sensitivity analysis

Sensitivity analysis will be conducted by excluding each qualified study. If the heterogeneity changes after excluding one literature, this literature may be the source of heterogeneity. Then, we will further analyze the reasons why this literature becomes the source of heterogeneity. If the heterogeneity remains unchanged after excluding each literature separately, our results are relatively robust.

### 2.9 Test of model fit

In the NMA, the established prediction model needs to be tested to compare the fitting degree between the predicted results and the actual situation. The “totresdev” calculated by WinBUGS software will be used to evaluate the fitting degree of the model. When the “totresdev” value is less than the total number of arms studied, the fitness of the model is good.

### 2.10 Assessment of publication bias and evidence quality

Where 10 or more trials per comparison are available, we will assess the potential publication bias using the Begg funnel plots. The quality of evidence derived from the network and pairwise meta-analysis will be appraised using the Grading of Recommendations Assessment, Development and Evaluation (GRADE) framework.^[32]^ The risk of bias, the indirectness of the evidence, inconsistency of the data, imprecision of effect estimates and publication bias will be measured.

## 3 Discussion

Approved by the National Drug Regulatory Authority, TCPM is processed according to prescribed prescription and preparation technology with Chinese herbal medicine as raw material, guided by the theory of TCM, including different dosage forms, such as pill, tablet, capsule, granule, et al. It is convenient to take and widely used with better therapeutic efficacy and fewer side effects. In our study, we will introduce NMA based on the existing RCTs to evaluate and rank the advantages and disadvantages of various TCMPs at the same time. We hope our findings are beneficial to alleviate the patient's suffering, for guideline development, and for Selection of clinical treatment programs. The quality of our analysis may be limited by the quality of the underlying data, such as possible publication bias in the eligible literature. Therefore, high-quality, multicenter clinical research and evidence are still needed to evaluate the efficacy and safety of TCPM for endometriosis in future studies.

## Author contributions

**Conceptualization:** Shuangqian Dong, Jianwei Zhang.

**Data curation**: Shuangqian Dong, Fengting Zhai, Jianwei Zhang.

**Methodology:** Shuangqian Dong, Jianwei Zhang, Xinglong Zhao, Xiuyun Qin.

**Project administration**: Xinglong Zhao.

**Search strategy**: Shuangqian Dong, Jianwei Zhang, Xiuyun Qin.

**Statistical analysis**: Shuangqian Dong, Fengting Zhai.

**Software:** Shuangqian Dong, Fengting Zhai, Xiuyun Qin.

**Writing – original draft**: Shuangqian Dong.

**Writing – review & editing:** Shuangqian Dong.
