# Comparative efficacy and safety of traditional Chinese patent medicine for endometriosis: A Bayesian network meta-analysis protocol: Erratum

**DOI:** 10.1097/MD.0000000000017264

**Published:** 2019-09-13

In the article, “Comparative efficacy and safety of traditional Chinese patent medicine for endometriosis: A Bayesian network meta-analysis protocol”,^[1]^ which appears in Volume 98, Issue 29 of *Medicine*, the affiliation a should appear as The First Clinical Medical College, Shandong University of Traditional Chinese Medicine, Jinan, Shandong Province.
